# A date pit induced aorto-oesophageal fistula: a case report and concise literature review

**DOI:** 10.1093/omcr/omaa140

**Published:** 2021-02-15

**Authors:** Kevin M Lichtenstein, Thomas B Russell, Julia B Lichtenstein, Harinderpal S Brar

**Affiliations:** 1 Department of Cardiac Surgery, Mazankowski Heart Institute, Edmonton, Alberta, Canada; 2 Department of General Surgery, University Hospitals Plymouth NHS Trust, Plymouth, Devon, UK; 3 Department of Plastic Surgery, Civic Hospital, Ottawa, Ontario, Canada; 4 Department of Critical Care, Surrey Memorial Hospital, Surrey, British Columbia, Canada

## Abstract

Aorto-oesophageal fistula (AEF) is rare and fatal without intervention. Having consumed a date pit 2 weeks prior, the patient in this case presented with the ‘Chiari’ triad of chest pain, sentinel arterial upper gastro-intestinal haemorrhage and exsanguination after an asymptomatic interval. Following resuscitation, the patient was managed with a Blakemore tube with both oesophageal and gastric balloons inflated to systemic pressures. An aortic stent graft was planned but the patient died on the operating table.

AEFs can be treated surgically with either open or endovascular repair. Open repair is highly risky and involves combined replacement/bypass of the thoracic aorta along with resection/repair of the involved oesophagus. Endovascular repair can prevent fatal exsanguination and increase the likelihood of survival but is associated with a significant rate of secondary infection, recurrence of fistula, mediastinitis and sepsis. Further studies are required to inform on management.

## INTRODUCTION

The following report details the case of a previously fit patient who developed an aorto-oesophageal fistula (AEF) following date pit consumption. This rare pathology most typically occurs following aneurysm of the descending thoracic aorta [1]. Foreign bodies account of about one-fifth of cases with button battery ingestion being the most frequently described [2]. Diagnosis is rarely made before massive haematemesis and it is rare for patients to survive [1]. Optimum management remains debated and is not evidence-based due to the low number of cases.

## CASE REPORT

A 76-year old female, previously fit and well, was admitted to hospital with new onset melaena. Two weeks prior she had accidentally swallowed a date pit. Seventy-two hours prior to admission she developed odynophagia and 24 h prior to admission she experienced a syncopal episode and hematemesis. She had no risk factors for gastro-intestinal bleeding. Following one night in hospital, the patient developed large volume haematemesis, hypotension and increased work of breathing. She was subsequently intubated due to airway compromise. A Blakemore tube was inserted, with both oesophageal and gastric balloons inflated, and traction applied. The haemorrhaging continued and a massive transfusion protocol was activated. The patient was referred to the intensive care unit (ICU) where she continued to haemorrhage profusely.

No obvious bleeding point was identified in the stomach or duodenum at endoscopy. A small, bleeding lesion was visualized on the oesophageal mucosa ~20 cm from the incisors. Attempts to control the haemorrhaging through localized adrenaline injection were unsuccessful. Computed tomography (CT) angiography was planned to identify a bleeding point. Profuse bleeding continued to the point of haemodynamic instability. The Blakemore tube was once again placed in the oesophagus and inflated to 30 mmHg. A total of 32 units of packed red blood cells, 20 units of fresh frozen plasma, three units of platelets and two units of cryoprecipitate were given. Control was eventually established by deflating the Blakemore tube and pulling it distally to the approximate site of the lesion. The oesophageal balloon was inflated to 100 mmHg and the gastric balloon was inflated to a lesser pressure.

**Figure 1:**
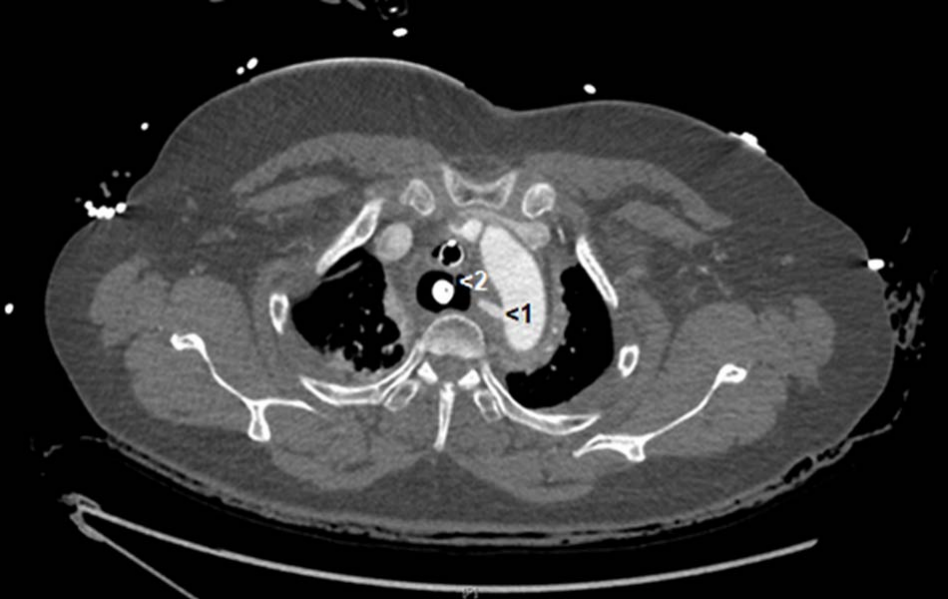
**Axial CT angiograph.** This image demonstrates the unusual arterial fistula (red number 1) connecting the arch of the aorta to the oesophagus. A Blakemore tube with inflated oesophageal balloon (red number 2), used to tamponade the arterial haemorrhage, can be seen within the oesophagus.

**Figure 2:**
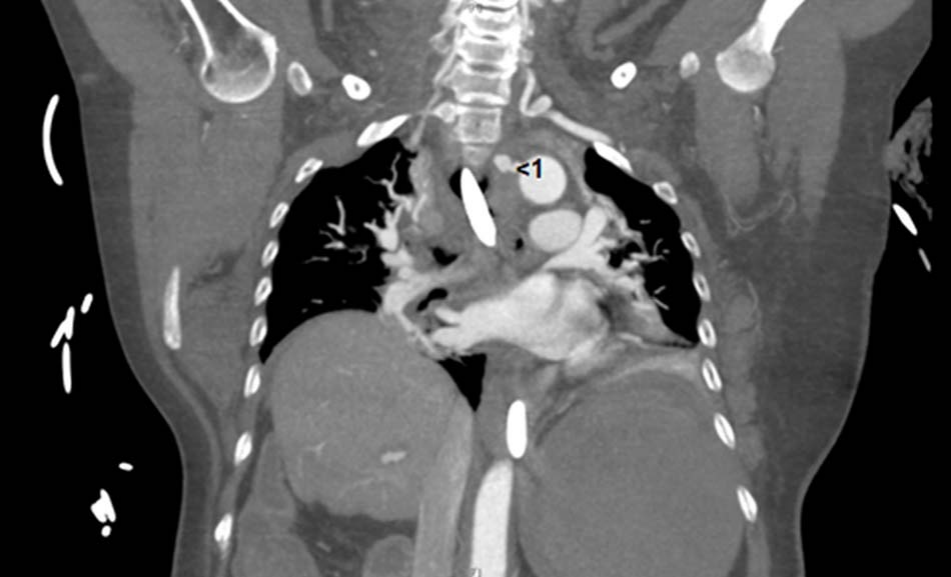
**Coronal CT angiograph.** This image demonstrates the unusual arterial fistula (red number 1) connecting the arch of the aorta to the oesophagus.

Computed tomography angiography (CTA) identified a large abnormal ‘vessel’ projecting from the medial aspect of the aortic arch extending towards the oesophageal wall (Figs 1 and 2). This structure did not have an aneurysmal configuration and was blind-ending (Figs 1 and 2). No discrete vascular ring was observed, and the anatomy of the great vessels was normal (Figs 1 and 2). Although tubular in nature, a mycotic aneurysm could not be ruled out. Takayasu’s arteritis was also considered. The atypical ‘vessel’ did not extend to the pulmonary artery as expected of a patent ductus arteriosus. Following discussion, it was concluded that the vascular abnormality was most likely a fistula tract between the oesophagus and aortic arch caused by a foreign body. Repair using a covered aortic stent graft was planned. During cannulation of the femoral artery the patient became acutely hypotensive and difficult to ventilate. She progressed rapidly to pulseless electrical activity cardiac arrest and died despite attempts at cardiopulmonary resuscitation.

## DISCUSSION

AEF is a rare, commonly fatal, cause of upper gastrointestinal bleeding where a new, abnormal communication between the oesophagus and aorta forms [1]. The swallowing of sharp, jagged objects, most commonly fish and chicken bones in adults, can lead to oesophageal and aortic perforation due to the close proximity of the two structures [2]. As in this case, foreign bodies or ulcerations causing fistulae are typically found 25–30 cm from the incisors where the oesophageal diameter narrows [3]. The cervical oesophagus, at the level of the cricopharyngeus, and the thoracic oesophagus, at the level of the aortic arch, are the two most common sites as this is where the aorta is in closest proximity [4].

The first reported case was in 1818 but treatment was not considered until the introduction of cardiopulmonary bypass [5]. The first successfully treated case was described in 1980 [3]. Patients with AEF are typically asymptomatic initially before presenting with the ‘Chiari triad’ of mid-thoracic pain, sentinel arterial haemorrhage and massive upper gastrointestinal bleeding [2]. Diagnosis is usually clinical and at least 45% of patients will present in this manner [1]. The sentinel bleed can precede massive haemorrhage by as little as a few minutes or as much as several weeks [5]. Eighty percent of patients present with a sentinel haemorrhage prior to massive haemorrhage [5]. Reports of dysphagia and odynophagia can be a forewarning for imminent massive haemorrhage [5]. Seventy-five percent of AEFs are due to aneurysm of the thoracic aorta with 9.7% of thoracic aortic aneurysms rupturing into the oesophagus [5]. Neoplastic processes, trauma, previous mediastinal surgery and aortic interventions are other causes [5].

As in this case, it is recommended that a Sengstaken-Blakemore tube be used to control haemorrhage until definitive surgical management occurs [6]. There have been no reported cases of survival from AEF with medical management alone [6]. Classical management of AEF was surgical correction with aortic cross clamping and cardiopulmonary bypass and simultaneous resection and repair of the thoracic oesophagus, although open repair has a 45–55% mortality [5]. Endovascular techniques have recently been used to treat AEF without the need for sternotomy and cardiopulmonary bypass [6]. In this case, thoracic endovascular aortic repair (TEVAR) was attempted. Previous studies have reported that TEVAR is a fast and safe method to treat AEF [7, 8]. It can obtain haemorrhage control acutely, reduce morbidity and mortality, and has high technical success rates [7]. However, TEVAR as a definitive procedure alone, versus as a short-term measure before open surgical repair, is still debated due to questions of durability [7]. While the minimally invasive technique provides the primary goal of therapy (rapid control of bleeding), the method does not address the digestive tract defect, and the graft is directly exposed to a contaminated environment. Thus, the risk for stent graft infection, fistula recurrence, persistent mediastinitis and sepsis is not insignificant [9]. A systematic review by Canaud *et al*. [7] demonstrated the presence of microorganisms in 43.2%, a recurrence rate of 13.8% and stent graft infection in 15.2% of the cases evaluated. The literature emphasizes the importance of long-term antibiotics in TEVAR cases to mitigate this risk, but some authors still stress that TEVAR alone cannot replace surgical debridement of the infected mediastinum [7, 9]. Long-term successful repair can be achieved using a combination of closed and open repair. Some authors describe TEVAR as a bridge therapy followed by a delayed open repair, once the patient is more stable, for debridement of the infected mediastinum, repair of the oesophageal defect, with or without stent graft exploration and reconstruction of the aortic wall [7, 10].
